# Complete Mitochondrial Genomes and Phylogenetic Positions of Two Longicorn Beetles, *Anoplophora glabripennis* and *Demonax pseudonotabilis* (Coleoptera: Cerambycidae)

**DOI:** 10.3390/genes13101881

**Published:** 2022-10-17

**Authors:** De-Qiang Pu, Hong-Ling Liu, Xing-Long Wu, Zhi-Teng Chen

**Affiliations:** 1 Key Laboratory of Integrated Pest Management of Southwest Crops, Institute of Plant Protection, Sichuan Academy of Agricultural Sciences, Chengdu 610066, China; 2 School of Grain Science and Technology, Jiangsu University of Science and Technology, Zhenjiang 212004, China

**Keywords:** Cerambycidae, mitogenome, longicorn beetle, gene arrangement, phylogeny

## Abstract

*Anoplophora glabripennis* (Motschulsky, 1854) and *Demonax pseudonotabilis* Gressitt & Rondon, 1970 are two commonly found longicorn beetles from China. However, the lack of sufficient molecular data hinders the understanding of their evolution and phylogenetic relationships with other species of Cerambycidae. This study sequenced and assembled the complete mitochondrial genomes of the two species using the next-generation sequencing method. The mitogenomes of *A. glabripennis* and *D. pseudonotabilis* are 15,622 bp and 15,527 bp in length, respectively. The mitochondrial gene content and gene order of *A. glabripennis* and *D. pseudonotabilis* are highly conserved with other sequenced longicorn beetles. The calculation of nonsynonymous (Ka) and synonymous (Ks) substitution rates in PCGs indicated the existence of purifying selection in the two longicorn beetles. The phylogenetic analysis was conducted using the protein-coding gene sequences from available mitogenomes of Cerambycidae. The two species sequenced in this study are, respectively, grouped with their relatives from the same subfamily. The monophyly of Cerambycinae, Dorcasominae, Lamiinae, and Necydalinae was well-supported, whereas Lepturinae, Prioninae, and Spondylidinae were recovered as paraphyletic.

## 1. Introduction

Cerambycidae (longicorn beetle) is one of the most speciose families of Coleoptera, comprising over 4000 genera and 35,000 species worldwide [1,2,3]. Cerambycidae *sensu stricto* (*s.s.*) usually consists of the eight subfamilies: Cerambycinae, Dorcasominae, Lamiinae, Lepturinae, Necydalinae, Parandrinae, Prioninae, and Spondylidinae [4]. Cerambycidae *sensu lato* (*s.l*.) comprises Cerambycidae *s.s.*, Disteniidae, Oxypeltidae, and Vesperidae [5]. The adults of longicorn beetles are morphologically diverse and phytophagous, usually feeding on living plant tissue, pollen, fruit, or tree sap [6]. Larvae of longicorn beetles usually have reduced or sometimes absent legs and they are mostly internal borers of their host plants [7,8,9,10]. In cultivated ecosystems, e.g., forest farms and tea gardens, the longicorn beetles are nonnegligible pests causing significant economic damage to the host plants [11,12].

The phylogeny and early evolution of Cerambycidae have been comprehensively reviewed by Haddad & Mckenna (2016) [13]. The phylogeny of longicorn beetles, especially the monophyly of Cerambycidae *s.s.* and *s.l.*, as well as the subfamily and tribe-level relationship, remains debatable due to the high species richness and highly variable morphological characters [5,14,15]. Haddad et al. (2018) [5] reconstructed the higher-level phylogeny of Cerambycidae with anchored hybrid enrichment of nuclear genes. Their results recovered a monophyletic Cerambycidae *s.s.* in most analyses and a polyphyletic Cerambycidae *s.l.* as well as the monophyletic subfamilies of Cerambycidae *s.s.* except for the paraphyletic Cerambycinae [5]. Nie et al. (2020) [15] used 151 mitochondrial genomes (mitogenomes) representing all families of Chrysomeloidea and all subfamilies of Cerambycidae *s.s.* to explore the higher-level phylogeny of Chrysomeloidea, especially Cerambycidae and allied families. However, their study could not support the monophyly of Cerambycidae *s.s.* and all its subfamilies. The two subfamilies, Necydalinae and Parandrinae, were considered as tribes Necydalini and Parandrini, respectively [15]. Meanwhile, the mitogenomes of many important cerambycid clades remained poorly represented, which restricted the accuracy of the results. 

The mitogenome is an informative molecular marker for taxonomic and evolutionary research and has become one of the most popular molecules used in current insect phylogenetic studies [16]. The development of next-generation sequencing techniques largely reduced the expense and experimental period to efficiently obtain the mitogenomes from all kinds of organisms. Diverse insect orders, such as Coleoptera [17,18], Lepidoptera [19], Hemiptera [20], etc., have combined the mitogenomes with dense taxon sampling to generate large-scale phylogenomic datasets for phylogenetic reconstruction and have revealed the strengths of mitogenomes in resolving the higher-level phylogenetic relationships. However, the available number of mitogenomes of Cerambycidae *s.l*. in the NCBI database is out of proportion to the remarkable species richness of longicorn beetles, which is a major impediment to better understanding the classification and evolution of this ecologically and economically significant group of insects.

To provide more genetic data for the longicorn beetles and investigate their phylogenetic relationships, this study sequenced and analyzed the mitogenomes of two commonly found longicorn beetles from China, *A. glabripennis* and *D. pseudonotabilis* [21,22]. Although the mitogenome of *A. glabripennis* (NC_008221) has been sequenced in a previous study [23], it is still very important to sequence more mitogenomes for the same species already listed in GenBank considering the existence of intraspecific variation of mitogenomes between different geographic populations [24]. Phylogenetic trees of Cerambycidae *s.l*. is constructed based on the newly sequenced as well as the known mitogenomic data to investigate the phylogenetic positions of the two newly sequenced species and provide more information for resolving the relationships within Cerambycidae *s.l*.

## 2. Materials and Methods

### 2.1. Sample Collection, DNA Extraction, and Mitogenome Sequencing

Adult specimens of *A. glabripennis* and *D. pseudonotabilis* were collected by Malaise traps set in the tea garden of Hongyan Town (29°59′31.42″ N, 103°10′34.45″ E), Mingshan County, Ya’an City, Sichuan Province of China, in 2016. The specimens were identified based on the morphological characteristics under a light microscope and were deposited in Sichuan Academy of Agricultural Sciences (specimen voucher: SAASCO1 (*A. glabripennis*) and SAASCO2 (*D. pseudonotabilis*)). All experiments and procedures for this study complied with the current animal ethics guidelines and did not involve any protected animals. 

The total genomic DNA was extracted by E.Z.N.A. Tissue DNA Kit (Omega, Norcross, GA, USA). At least 1 µg of purified DNA was used to construct the TruSeq DNA library with an insert size of 400 bp according to standard protocols. The library was sequenced using the Illumina HiSeq 4000 platform (Personal Gene Technology Co., Ltd., Nanjing, China) with paired-end reads of 2 × 150 bp. A total of 21,616,708 and 22,096,010 raw reads were obtained for *A. glabripennis* and *D. pseudonotabilis*, respectively. Over 97.8% of bases in the raw reads were regarded as correctly identified with an accuracy rate above 99%. The unpaired, short, and low-quality raw reads were filtered by fastp [25] to obtain clean reads. The above quality-control and data-filtering process generated 21,584,444 and 22,059,322 high-quality reads for *A. glabripennis* and *D. pseudonotabilis*, respectively.

### 2.2. Mitogenome Assembly, Annotation, and Analyses

Before the assembly, the high-quality reads were trimmed again using BBDuk with default settings implemented in Geneious Prime [26]. The high-quality reads of *A. glabripennis* and *D. pseudonotabilis* were, respectively, mapped to the reference mitogenome of the previously sequenced *A. glabripennis* (NC_008221) [23] and amplified bilaterally by Geneious Prime [26], with the parameters set as follows: 95% minimum overlap identity, 50 bp minimum overlap, and maximum ambiguity as 4. The completeness of each circular mitogenome was confirmed when both ends of the final assembled contigs overlapped (100% coverage). The assembled mitogenomes of *A. glabripennis* and *D. pseudonotabilis* were deposited in GenBank under the accession numbers OP096420 and OP096419, respectively. 

The two mitogenomes were annotated in the MITOS web server [27]. The resultant gene boundaries of the protein-coding genes (PCGs) were checked manually by the NCBI’s ORF Finder (https://www.ncbi.nlm.nih.gov/orffinder/, accessed on 9 August 2022). The location and secondary structures of the transfer RNA (tRNA) and ribosomal RNA (rRNA) genes were predicted and visualized by MITOS. The mitogenome structure and GC skews were visualized by the CGView Server [28]. The nucleotide composition, skews, codon usage, and relative synonymous codon usage (RSCU) were calculated by MEGA11 [29]. The synonymous substitution rate (Ks) and nonsynonymous substitution rate (Ka) were calculated using KaKs_Calculator v2.0, with the mitogenome of *Aoria nigripes* (Baly, 1860) (Chrysomelidae) as the outgroup [30,31]. The alignment file of each PCG was uploaded to the Datamonkey web server for a more thorough exploration of selective pressure in PCGs of the two newly sequenced mitogenomes. BUSTED (Branch-Site Unrestricted Statistical Test for Episodic Diversification) was used to test whether each PCG has experienced positive selection [32]. FEL (fixed-effects likelihood) was employed to infer site-specific Ks and Ka values and detect the following four types of sites in each PCG: diversifying sites, purifying sites, neutral sites, and invariable sites [33]. Tandem repeats in the control regions were identified using the Tandem Repeats Finder web server [34]. The stem-loop structures in the control region were predicted by the Mfold web server with default settings [35].

### 2.3. Phylogenetic Analyses Methods

The phylogenetic relationships were reconstructed based on the nucleotide sequences of 13 PCGs derived from 186 mitogenomes of Cerambycidae *s.l.* (Table 1). Overall, 30 of the 186 mitogenomes were originally unannotated in GenBank; they were re-annotated by MITOS and manual homology alignments in this study. Other mitogenomes from GenBank that had incomplete set of 13 PCGs or incorrect PCG sequences were omitted from the dataset. The mitogenome of *A. nigripes* (Chrysomelidae) was used as the outgroup [31]. The 13 PCGs were, respectively, aligned using MUSCLE with a codon mode [36], followed by the deletion of stop codons and the concatenation of sequences by SequenceMatrix v1.7.8 [37]. The best-fit partitioning schemes and substitution models for each PCG region were determined by PartitionFinder v2.1.1 using the Bayesian information criterion (BIC) and a greedy search algorithm of all available models [38]. Phylogenies were inferred using maximum-likelihood (ML) and Bayesian inference (BI) methods. The best-fit model was GTR+I+G for two partitioned subsets: one subset included *ND1*, *ND4*, *ND4L*, and *ND5*; the other subset included the remaining 9 PCGs. IQ-Tree was used to perform the ML analysis under the edge-unlinked partition model for 5000 ultrafast bootstraps as well as the Shimodaira–Hasegawa-like approximate likelihood-ratio test [39,40,41]. The BI analysis was conducted by MrBayes v3.2.7 [42] with four independent Markov chains for 30 million generations and sampled every 100 generations. The first 25% of the trees were discarded as burn-in. FigTree v1.4.4 was used to edit and visualize the phylogenetic trees [43].

## 3. Results and Discussion

### 3.1. Genome Structure and Composition

**Figure 1:**
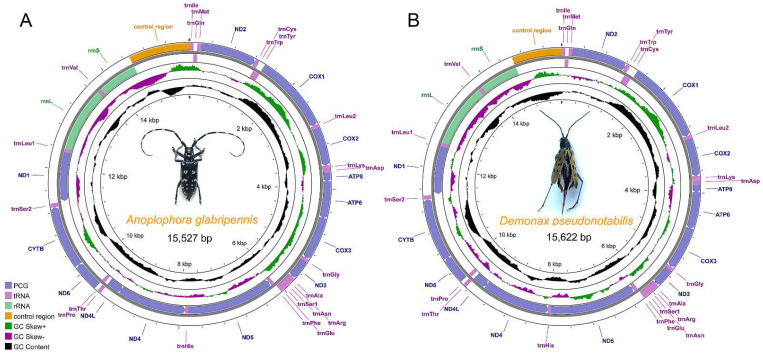
Mitochondrial genome maps of *A. glabripennis* (**A**) and *D. pseudonotabilis* (**B**). Genes outside the map are transcribed clockwise, whereas those inside the map are transcribed counterclockwise. The inside circles show the GC content and the GC skew. GC content and GC skew are plotted as the deviation from the average value of the entire sequence.

The assembled complete mitogenomes of *A. glabripennis* and *D. pseudonotabilis* are circular DNA molecules of 15,622 bp and 15,527 bp in length (Figure 1), respectively, which is within the range of the sequenced mitogenomes of Cerambycidae in GenBank (Table 1). Due to the presence of a shorter *COX1* gene, the newly obtained *A. glabripennis* mitogenome is slightly shorter than the previously sequenced mitogenome (15,774 bp) based on samples from Hebei Province [31]. Both newly sequenced mitogenomes contain the standard set of 37 mitochondrial genes (13 PCGs, 22 tRNA genes, and 2 rRNA genes) as all other longicorn beetles. The gene order is identical to all other species of Cerambycidae as well as the ancestral mitogenome type of *Drosophila yakuba* Burla, 1954 [14,44,45]. Among the 37 genes, 23 (9 PCGs and 14 tRNAs) genes are on the majority strand (J-strand), while the remaining 4 PCGs, 8 tRNAs, and 2 rRNA genes are on the minority strand (N-strand). 

A total of nine gene overlapping regions were found in the *A. glabripennis* mitogenome with a total of 29 bp in length, and the longest overlapping sequence (8 bp) was located between *trnCys* and *trnTyr*. In the *D. pseudonotabilis* mitogenome, there are 12 overlapping regions with a total of 21 bp in length, and the longest overlapping sequences were only 4 bp in length. The universally found 7 bp overlapping regions between *ATP8* and *ATP6*, as well as *NAD4* and *NAD4L* in Cerambycidae and many other insects [14,15], are restricted to the overlapping between *NAD4* and *NAD4L* in the *A. glabripennis* mitogenome, which might be resulted from the different annotation methods. In addition to the overlapping regions, multiple intergenic spacers are scattered throughout both mitogenomes (Table 2 and Table 3). The base composition is 38.8% A, 14.2% C, 9.2% G, and 37.8% T for the *A. glabripennis* mitogenome and 39.7% A, 14.5% C, 10.5% G, and 35.3% T for *D. pseudonotabilis*. The two mitogenomes are highly skewed towards A and T nucleotides, with an A + T content of 76.6% in *A. glabripennis* and 75.0% in *D. pseudonotabilis* (Table 1).

### 3.2. Protein-Coding Genes

The PCGs have identical arrangement and similar size between the two mitogenomes and also other cerambycids. Most PCGs of the two species start with the standard ATN start codons (ATA, ATC, ATG, and ATT), whereas *ND1* of both mitogenomes begins with the special codon TTG (Table 2 and Table 3), which was similar to all other published Cerambycidae mitogenomes [14,15]. Most PCGs of each mitogenome have the complete termination codon TAN (TAA, TAT, or TAG), whereas four PCGs (*COX1*, *COX2*, *ND4*, and *ND5*) of *A. glabripennis* and four PCGs (*COX1*, *COX3*, *ND3*, and *ND5*) of *D. pseudonotabilis* end with an incomplete stop codon T. These incomplete stop codons are considered to be caused by the post-transcriptional polyadenylation [46] and can be completed by the addition of 3′ nucleotide residues to the neighboring mitochondrial genes.

**Figure 2:**
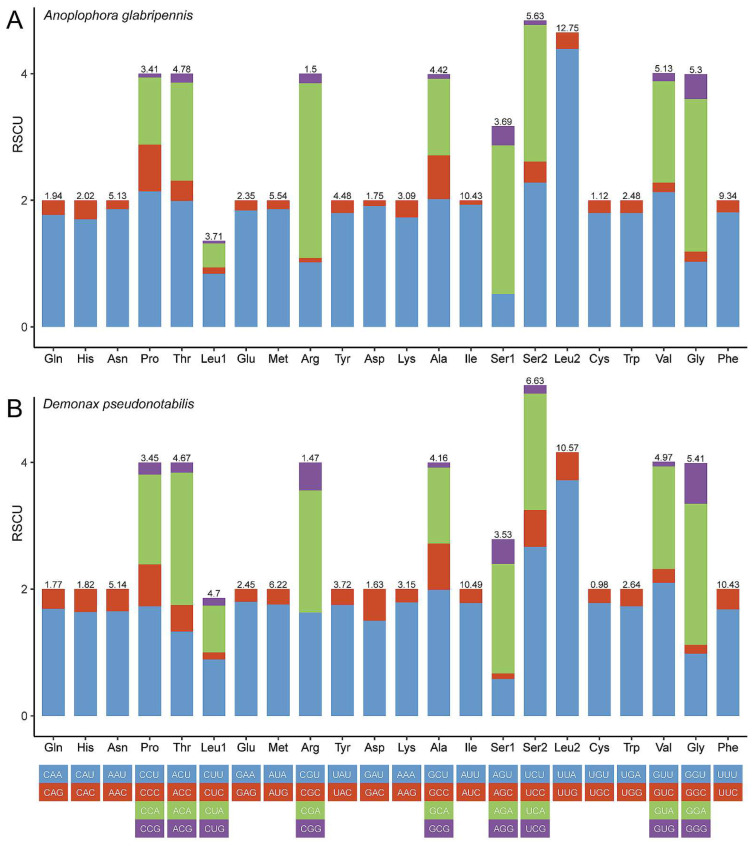
Relative synonymous codon usage (RSCU) of PCGs in *A. glabripennis* (**A**) and *D. pseudonotabilis* (**B**).

**Figure 3:**
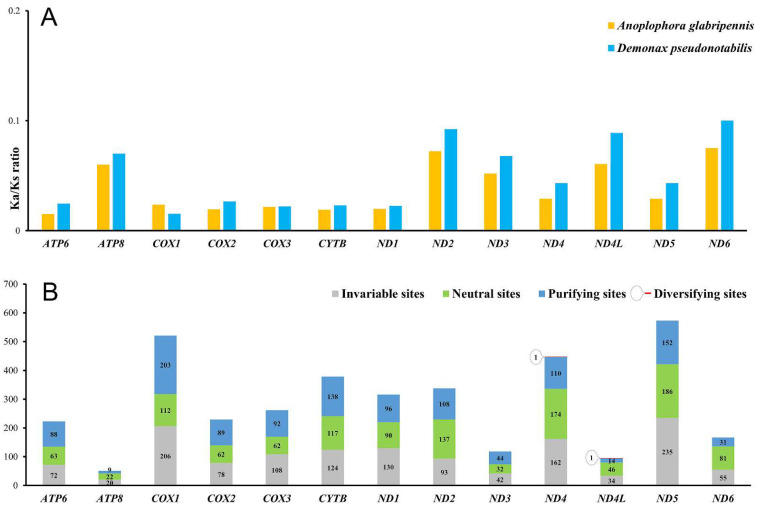
Nonsynonymous/synonymous substitution ratios (**A**) and codon sites diversity (**B**) of mitochondrial PCGs of *A. glabripennis* and *D. pseudonotabilis*.

The relative synonymous codon usage (RSCU) values indicate the most frequently used codon is TTA (Leu) for both mitogenomes (Figure 2), which appears to be a common feature of other sequenced longicorn beetles [14]. *ATP8* of both mitogenomes has the highest A + T content among the 13 PCGs (Table 2 and Table 3). The Ka/Ks ratios for each PCG of each mitogenome are calculated to assess the selective pressure of the two cerambycid species (Figure 3A). The evolutionary rate of *ND6* was the highest among the 13 PCGs. The Ka/Ks ratios of all the 13 PCGs calculated by KaKs_Calculator v2.0 were below 1, which suggests the existence of purifying selection in the two species (Figure 3A). The results of Ka/Ks calculation were similar to a recent mitogenomic work [47], which used DnaSP for the calculation. The gene-wide BUSTED analysis based on the likelihood-ratio test found no evidence of episodic diversifying selection in the PCGs. The site-specific FEL analysis detected *ND4* and *ND4L* each had one codon site under diversifying positive selection at *p* ≤ 0.1 (Figure 3B). Nearly one-third of each PCG’s codon sites were under purifying selection at *p* ≤ 0.1. The calculation of KaKs_Calculator v2.0 was consistent with the results of FEL analysis that the PCGs with lower Ka/Ks ratios tended to have more purifying codon sites (Figure 3). 

### 3.3. Transfer RNAs, Ribosomal RNAs, and Control Region

**Figure 4:**
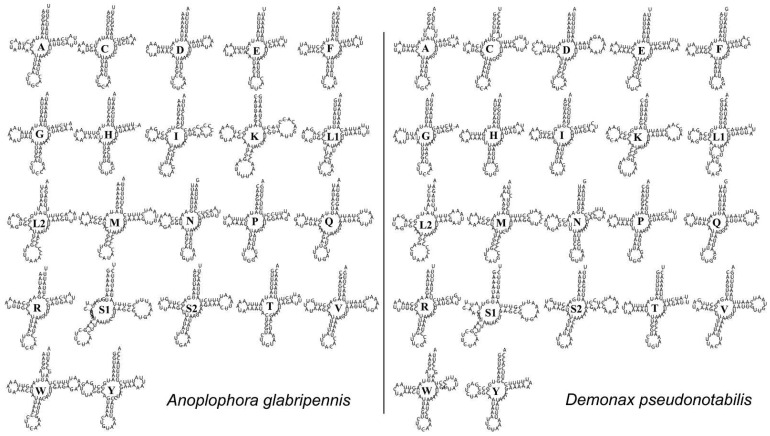
Secondary structures of tRNA genes in the mitogenomes of *A. glabripennis* and *D. pseudonotabilis*. The identity of each tRNA gene is represented by the abbreviation of the related amino acid.

The two mitogenomes both contain the complete set of 22 tRNA genes typical of metazoan mitogenomes. These tRNAs range in size from 62 to 69 bp, which was consistent with previously sequenced mitogenomes of Cerambycidae [15]. The highest A + T content is found in *trnGlu* of both mitogenomes (Table 2 and Table 3). Most of the tRNAs have typical cloverleaf secondary structures, whereas the dihydrouridine (DHU) arm of *trnSer1* is shortened in both mitogenomes (Figure 4), which is a common phenomenon in hexapods and metazoan mitogenomes [48]. Numerous mismatched base pairs are found in the secondary structures of tRNA genes, and all of them are G–U pairs.

The large ribosomal RNA (*rrnL*) gene and small ribosomal RNA (*rrnS*) gene are found in the conserved location between *trnLeu1* and the control region (Table 2 and Table 3). The *rrnL* gene is 1272 bp long in *A. glabripennis* and 1266 bp long in *D. pseudonotabilis*, with an A + T content of 80.1% and 78.8%, respectively. The *rrnS* gene is 779 bp long in *A. glabripennis* and 774 bp long in *D. pseudonotabilis*, with an A + T content of 78.6% and 76.5%, respectively. 

The control region (CR) is the longest non-coding area in the two mitogenomes (Figure 1) and is functional in the regulation, transcription, and replication processes of the mitogenomes [49]. The CR of *A. glabripennis* is 1104 bp long and has an A + T content of 79.3%; the CR of *D. pseudonotabilis* is 907 bp long and has an A + T content of 82.1% (Table 2 and Table 3). In the CR of *A. glabripennis*, 5.2 copies of 57 bp long tandem repeat “AAAATTTCATCAGCTAGCTCCGCTATATAAAATCGCCTACCTTTCAAATTTCCCCTA” are detected near the 5′ end of this region. A total of 22 standard (single stem with single loop) and another 7 more complicated stem-loop structures are predicted in the CR of *A. glabripennis* (Figure S1). There are 17 standard and 4 complicated stem-loop structures in the CR of *D. pseudonotabilis* (Figure S2). However, no tandem repeats are found in the CR of *D. pseudonotabilis*. Functions of these secondary structures are unclear.

### 3.4. Phylogenetic Analyses

**Figure 5:**
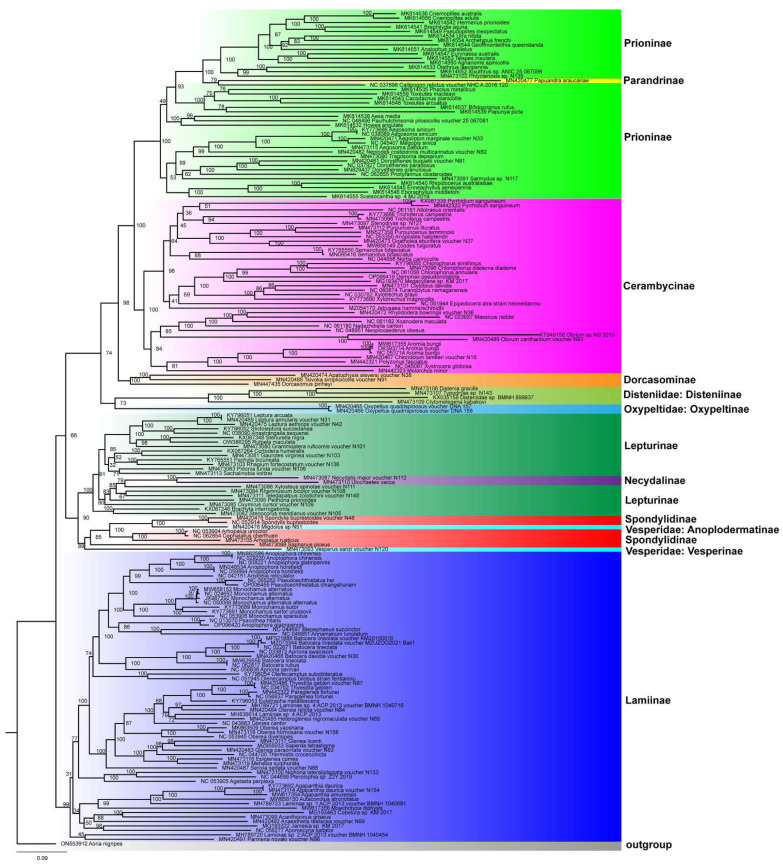
Maximum-likelihood phylogeny of Cerambycidae *s.l.* inferred from mitogenomic data. Numbers at the nodes are bootstrap values.

The phylogenetic positions of *A. glabripennis* and *D. pseudonotabilis* are reconstructed based on the combined mitochondrial gene set of 13 PCGs. The ML and BI analyses generated similar tree topology (Figure 5 and Figure S3). The phylogenetic results are largely congruent with the recent comprehensive mitogenomic phylogenetic study of Nie et al. (2020) [15]. The monophyly of Cerambycidae *s.s.* is not well-supported in both ML and BI trees due to the inclusion of other families of Chrysomeloidea (Figure 5), which is similar to the results of Haddad et al. (2018) [5] and Nie et al. (2020) [15]. The positions of Disteniidae and Oxypeltidae are variable, and Oxypeltidae is recovered as the sister group to all other taxa in the BI tree (Figure S3). The phylogenetic position of Disteniidae remains uncertain, and this family has been recovered as the sister group to various other members of Cerambycidae *s.l.* based on either molecular or morphological datasets [5,9,15,50,51,52,53,54,55,56,57]. The monophyly of Cerambycidae *s.s.* is still one of the most debatable subjects in the phylogeny and evolution of Chrysomeloidea [5]. 

The monophyly of Cerambycinae, Dorcasominae, Lamiinae, and Necydalinae is well-supported in both ML and BI analyses (Figure 5 and Figure S3). The subfamily Parandrinae is placed within Prioninae and should be treated as a tribe of Prioninae, as suggested in previous studies [15,58]. Similarly, Necydalinae is nested in Lepturinae and should be regarded as a tribe of Lepturinae [15,50]. Spondylidinae is rendered paraphyletic by the species of Vesperidae, which differs from the monophyletic condition in Haddad et al. (2018) [5] and Nie et al. (2020) [15]. The non-monophyletic condition of Vesperidae has also been recovered based on morphological and molecular characters [54,55,59,60,61]. The two species sequenced in this study are, respectively, grouped with their relatives from the same subfamily.

Although numerous contributions have been made to explore the higher-level phylogeny of longhorn beetles, there are still some debatable points to be solved: the monophyly of Cerambycidae *s.l.* and *s.s.*; the relative relationship between Cerambycidae *s.s.*, Disteniidae, Oxypeltidae, and Vesperidae; and the monophyly and relationship of subfamilies in Cerambycidae *s.l.*, especially within Cerambycidae *s.s*. The incongruence between different molecular phylogenetic studies could be attributed to the usage of different molecular types, sample sizes, and analytical methods. The taxonomic misidentification of sequenced samples in online databases such as GenBank could also lead to bizarre tree topology, especially for those clades with few taxa. Most main clades of Cerambycidae *s.l.* still lack sufficient molecular data to clarify their phylogenetic positions. The sequencing of more mitogenomes, optimization of datasets and substitution models, and the supplement of nuclear genes are expected to improve the resolution of mitochondrial phylogenetic reconstruction of Cerambycidae *s.l.* in future works.

## 4. Conclusions

In this study, we sequenced and analyzed the mitogenomes of two longicorn beetles, which are important pests of cultivated ecosystems in China. The structure and content of the two mitogenomes are conserved in comparison to other sequenced mitogenomes of Cerambycidae, but the intraspecific mitogenomic variation is also detected. The monophyly of four subfamilies was supported by the phylogenetic analysis based on the nucleotide sequence of PCGs. The results provided basic genetic information for understanding the phylogeny and evolution of longicorn beetles.

## Figures and Tables

**Table 1:** Species used in this study.

Family	Subfamily	Species	Genome Size (bp)	GenBank No.
Cerambycidae *s.s.*	Cerambycinae	*Allotraeus orientalis*	15,966	NC_061181
		*Anoplistes halodendri*	15,697	NC_053350
		*Aromia bungii*	15,652	MW617355
		*A. bungii*	15,760	NC_053714
		*A. bungii*	15,759	OK393714
		*Chloridolum lameeri*	15,731	MN420467
		*Chlorophorus annularis*	15,487	NC_061058
		*Chlorophorus diadema*	15,398	MN473096
		*Chlorophorus simillimus*	13,675	KY796055
		*Clytobius davidis*	15,571	MN473101
		*D. pseudonotabilis*	15,527	OP096419
		*Epipedocera atra*	15,662	NC_051944
		*Gnatholea eburifera*	15,281	MN420473
		*Jebusaea hammerschmidtii*	15,619	MZ054170
		*Massicus raddei*	15,858	NC_023937
		*Megacyllene* sp. KM-2017	15,832	MG193470
		*Molorchus minor*	15,685	MN442323
		*Nadezhdiella cantori*	16,049	NC_061180
		*Neoplocaederus obesus*	15,683	NC_048951
		*Nortia carinicollis*	15,602	NC_044698
		*Obrium cantharinum*	15,632	MN420489
		*Obrium* sp. NS-2015	15,680	KT945156
		*Polyzonus fasciatus*	15,804	MN442321
		*Purpuricenus lituratus*	15,744	MN473112
		*Purpuricenus temminckii*	15,689	MN527358
		*Pyrrhidium sanguineum*	16,203	KX087339
		*P. sanguineum*	15,748	MN442320
		*Rhytidodera bowringii*	15,278	MN420472
		*Semanotus bifasciatus*	13,837	KY765550
		*S. bifasciatus*	16,051	MN095416
		*Stenodryas* sp. N127	15,333	MN473097
		*Trichoferus campestris*	13,696	KY773688
		*T. campestris*	15,737	MN473098
		*Turanoclytus namaganensis*	15,565	NC_060874
		*Xoanodera maculata*	15,767	NC_061182
		*Xylotrechus grayii*	15,540	NC_030782
		*Xylotrechus magnicollis*	13,692	KY773690
		*Xystrocera globosa*	15,707	NC_045097
		*Zoodes fulguratus*	15,885	MW858149
	Dorcasominae	*Apatophysis sieversi*	15,278	MN420474
		*Dorcasomus pinheyi*	16,040	MN447435
		*Tsivoka simplicicollis*	16,700	MN420488
	Lamiinae	*Acanthocinus griseus*	15,600	MN473099
		*Agapanthia amurensis*	15,512	MW617354
		*Agapanthia daurica*	14,282	KY773692
		*A. daurica*	17,153	MN473114
		*Agelasta perplexa*	15,552	NC_053905
		*Anaesthetis testacea*	15,169	MN420492
		*Annamanum lunulatum*	15,610	NC_046851
		*Anoplophora chinensis*	15,871	MN882586
		*A. chinensis*	15,805	NC_029230
		*A. glabripennis*	15,774	NC_008221
		*Anoplophora horsfieldi*	15,796	MN248534
		*A. horsfieldi*	15,837	NC_059864
		*A. glabripennis*	15,622	OP096420
		*Apomecyna saltator*	14,949	NC_056277
		*Apriona germarii*	14,858	NC_056838
		*Apriona swainsoni*	15,412	NC_033872
		*Aristobia reticulator*	15,838	NC_042151
		*Aulaconotus atronotatus*	14,491	MW858150
		*Batocera davidis*	15,554	MN420468
		*Batocera lineolata*	15,420	MF521888
		*B. lineolata*	16,158	MW629558
		*B. lineolata*	15,420	MZ073344
		*B. lineolata*	15,418	NC_022671
		*Batocera rubus*	16,158	NC_062817
		*Blepephaeus succinctor*	15,554	NC_044697
		*Cobelura* sp. KM-2017	15,912	MG193463
		*Epiglenea comes*	15,213	MN473116
		*Eutetrapha metallescens*	15,072	KY796053
		*Glenea cantor*	15,514	NC_043883
		*Glenea licenti*	15,435	MN473117
		*Glenea paraornata*	15,510	MN420483
		*Glenea relicta*	15,486	MN420484
		*Heteroglenea nigromaculata*	15,502	MN420485
		*Jamesia* sp. KM-2017	17,430	MG193322
		*Lamiinae* sp. 1 ACP-2013	15,737	MH789723
		*Lamiinae* sp. 2 ACP-2013	15,440	MH789720
		*Lamiinae* sp. 4 ACP-2013	15,504	MH789721
		*Lamiinae* sp. 4 ACP-2013	15,554	MH836614
		*Menesia sulphurata*	15,551	MN473119
		*Moechotypa diphysis*	15,493	MW617356
		*Monochamus alternatus*	14,649	JX987292
		*M. alternatus*	14,189	MW858152
		*M. alternatus*	15,874	NC_024652
		*M. alternatus*	15,880	NC_050066
		*Monochamus sartor urussovii*	14,359	KY773691
		*Monochamus sparsutus*	16,029	NC_053906
		*Monochamus sutor*	14,350	KY773689
		*Niphona lateraliplagiata*	15,902	MN473100
		*Oberea diversipes*	15,499	NC_053945
		*Oberea formosana*	15,675	MN473118
		*Oberea yaoshana*	15,529	MK863509
		*Olenecamptus bilobus*	15,262	NC_051945
		*Olenecamptus subobliteratus*	13,854	KY796054
		*Paraglenea fortunei*	15,401	MN442322
		*P. fortunei*	15,496	NC_056837
		*Parmena novaki*	15,668	MN420491
		*Psacothea hilaris*	15,856	NC_013070
		*Pseudoechthistatus chiangshunani*	16,419	OP006455
		*Pseudoechthistatus hei*	16,103	NC_065262
		*Pterolophia* sp. ZJY-2019	16,063	NC_044699
		*Saperda tetrastigma*	15,563	MZ955033
		*Serixia sedata*	14,714	MN420487
		*Thermistis croceocincta*	15,503	NC_044700
		*Thyestilla gebleri*	15,503	MN420486
		*T. gebleri*	15,505	NC_034752
	Lepturinae	*Anastrangalia sequensi*	16,269	NC_038090
		*Brachyta interrogationis*	18,165	KX087246
		*Cortodera humeralis*	15,928	KX087264
		*Gaurotes virginea*	15,775	MN473081
		*Grammoptera ruficornis*	16,458	MN473080
		*Leptura aethiops*	15,690	MN420475
		*Leptura annularis*	16,530	MN420469
		*Leptura arcuata*	14,382	KY796051
		*Oxymirus cursor*	15,797	MN473085
		*Pachyta bicuneata*	13,894	KY765551
		*Peithona prionoides*	13,636	MN473095
		*Pidonia lurida*	15,668	MN473083
		*Rhagium fortecostatum*	16,274	MN473103
		*Rhamnusium bicolor*	15,527	MN473084
		*Rutpela maculata*	17,437	OW386295
		*Sachalinobia koltzei*	15,809	MN473113
		*Stenurella nigra*	16,504	KX087348
		*Stictoleptura succedanea*	14,381	KY796052
		*Teledapalpus zolotichini*	16,651	MN473111
		*Stenocorus meridianus*	16,227	MN473082
		*Xylosteus spinolae*	15,708	MN473086
	Necydalinae	*Necydalis major*	15,598	MN473087
		*Ulochaetes vacca*	15,593	MN473110
	Parandrinae	*Papuandra araucariae*	15,475	MN420477
	Prioninae	*Aegolipton marginale*	16,759	MN420471
		*Aegosoma pallidum*	15,668	MN473115
		*Aegosoma sinicum*	15,658	KY773686
		*A. sinicum*	15,658	NC_038089
		*Aesa media*	15,714	MK614538
		*Agrianome spinicollis*	15,633	MK614550
		*Analophus parallelus*	15,722	MK614551
		*Archetypus frenchi*	16,156	MK614554
		*Bifidoprionus rufus*	15,590	MK614537
		*Brephilydia jejuna*	15,659	MK614541
		*Cacodacnus planicollis*	15,671	MK614543
		*Callipogon relictus*	15,742	NC_037698
		*Cnemoplites australis*	15,675	MK614536
		*Cnemoplites edulis*	13,161	MK614556
		*Dorysthenes buquetii*	15,778	MN420481
		*Dorysthenes granulosus*	15,858	MN829437
		*Dorysthenes paradoxus*	15,922	NC_037927
		*Eboraphyllus middletoni*	15,776	MK614546
		*Enneaphyllus aeneipennis*	16,505	MK614545
		*Eurynassa australis*	15,612	MK614547
		*Geoffmonteithia queenslanda*	15,628	MK614544
		*Hermerius prionoides*	13,696	MK614542
		*Howea angulata*	15,626	MK614532
		*Megopis sinica*	15,689	NC_045407
		*Nepiodes costipennis multicarinatus*	15,935	MN420482
		*Olethrius laevipennis*	15,690	MK614533
		*Papunya picta*	15,737	MK614539
		*Paulhutchinsonia pilosicollis*	15,846	NC_048496
		*Phaolus metallicus*	15,997	MK614535
		*Phlyctenosis* sp. N135	15,000	MN473102
		*Priotyrannus closteroides*	15,854	NC_062855
		*Pseudoplites inexpectatus*	15,651	MK614549
		*Rhipidocerus australasiae*	15,721	MK614540
		*Sarmydus* sp. N117	15,720	MN473091
		*Sceleocantha* sp. 4 MJ-2019	15,804	MK614555
		*Teispes insularis*	15,632	MK614553
		*Toxeutes arcuatus*	15,859	MK614548
		*Toxeutes macleayi*	13,579	MK614559
		*Tragosoma depsarium*	15,712	MN473090
		*Utra nitida*	14,976	MK614534
		*Xixuthrus* sp. ANIC_25-067096	15,523	MK614552
	Spondylidinae	*Arhopalus rusticus*	15,860	MN473105
		*Arhopalus unicolor*	15,760	NC_053904
		*Cephalallus oberthueri*	15,763	NC_062854
		*Saphanus piceus*	15,832	MN473088
		*Spondylis buprestoides*	16,070	MN420476
		*S. buprestoides*	15,837	NC_052914
Disteniidae	Disteniinae	*Clytomelegena kabakovi*	15,816	MN473109
		*Distenia gracilis*	15,704	MN473106
		*Disteniinae* sp. BMNH 899837	15,598	KX035158
		*Typodryas* sp. N143	15,647	MN473107
Oxypeltidae	Oxypeltinae	*Oxypeltus quadrispinosus*	16,140	MN420465
		*O. quadrispinosus*	17,001	MN420466
Vesperidae	Anoplodermatinae	*Migdolus* sp. N51	14,931	MN420478
	Vesperinae	*Vesperus sanzi*	16,125	MN473093
Chrysomelidae		*A. nigripes*	17,306	ON553912

**Table 2:** Mitochondrial genome organization of *A. glabripennis*.

Gene	Position (bp)	Size (bp)	Direction	Intergenic Nucleotides	Anti− or Start/Stop Codons	A + T%
*trnIle (I)*	1–67	67	Forward	0	GAT	61.2
*trnGln (Q)*	69–137	69	Reverse	1	TTG	78.3
*trnMet (M)*	137–205	69	Forward	−1	CAT	72.5
*ND2*	206–1216	1011	Forward	0	ATT/TAA	77.6
*trnTrp (W)*	1215–1282	68	Forward	−2	TCA	76.5
*trnCys (C)*	1275–1336	62	Reverse	−8	GCA	74.2
*trnTyr (Y)*	1338–1402	65	Reverse	1	GTA	69.2
*COX1*	1403–2819	1417	Forward	0	ATC/T	68.1
*trnLeu2 (L2)*	2820–2884	65	Forward	0	TAA	73.8
*COX2*	2885–3572	688	Forward	0	ATC/T	72.1
*trnLys (K)*	3573–3641	69	Forward	0	CTT	68.1
*trnAsp (D)*	3642–3707	66	Forward	0	GTC	86.4
*ATP8*	3708–3863	156	Forward	0	ATT/TAG	86.5
*ATP6*	3860–4531	672	Forward	−4	ATA/TAA	75.1
*COX3*	4531–5319	789	Forward	−1	ATG/TAA	70.6
*trnGly (G)*	5322–5385	64	Forward	2	TCC	85.9
*ND3*	5383–5739	357	Forward	−3	ATA/TAG	79.0
*trnAla (A)*	5738–5802	65	Forward	−2	TGC	81.5
*trnArg (R)*	5803–5864	62	Forward	0	TCG	74.2
*trnAsn (N)*	5864–5927	64	Forward	−1	GTT	75.0
*trnSer1 (S1)*	5928–5994	67	Forward	0	GCT	76.1
*trnGlu (E)*	5995–6057	63	Forward	0	TTC	87.3
*trnPhe (F)*	6060–6123	64	Reverse	2	GAA	82.8
*ND5*	6124–7840	1717	Reverse	0	ATT/T	78.3
*trnHis (H)*	7841–7903	63	Reverse	0	GTG	84.1
*ND4*	7904–9236	1333	Reverse	0	ATG/T	79.3
*ND4L*	9230–9517	288	Reverse	−7	ATG/TAA	83.0
*trnThr (T)*	9520–9583	64	Forward	2	TGT	82.8
*trnPro (P)*	9584–9647	64	Reverse	0	TGG	78.1
*ND6*	9650–10,153	504	Forward	2	ATT/TAA	85.1
*CYTB*	10,159–11,292	1134	Forward	5	ATA/TAA	72.2
*trnSer2 (S2)*	11,296–11,364	69	Forward	3	TGA	81.2
*ND1*	11,382–12,332	951	Reverse	17	TTG/TAG	76.3
*trnLeu1 (L1)*	12,334–12,398	65	Reverse	1	TAG	78.5
*rrnL*	12,399–13,670	1272	Reverse	0		80.1
*trnVal (V)*	13,671–13,739	69	Reverse	0	TAC	75.4
*rrnS*	13,740–14,518	779	Reverse	0		78.6
Control Region	14,519–15,622	1104	Forward	0		79.3

**Table 3:** Mitochondrial genome organization of *D. pseudonotabilis*.

Gene	Position (bp)	Size (bp)	Direction	Intergenic Nucleotides	Anti− or Start/Stop Codons	A + T%
*trnIle (I)*	1–66	66	Forward	0	GAT	72.7
*trnGln (Q)*	64–132	69	Reverse	−3	TTG	81.2
*trnMet (M)*	132–200	69	Forward	−1	CAT	65.2
*ND2*	201–1211	1011	Forward	0	ATA/TAA	76.2
*trnTrp (W)*	1210–1274	65	Forward	−2	TCA	73.8
*trnCys (C)*	1274–1339	66	Reverse	−1	GCA	72.7
*trnTyr (Y)*	1341–1405	65	Reverse	1	GTA	66.2
*COX1*	1440–2940	1501	Forward	34	ATT/T	67.0
*trnLeu2 (L2)*	2941–3005	65	Forward	0	TAA	72.3
*COX2*	3006–3692	687	Forward	0	ATA/TAT	70.7
*trnLys (K)*	3694–3764	71	Forward	1	CTT	70.4
*trnAsp (D)*	3768–3837	70	Forward	3	GTC	82.9
*ATP8*	3847–3993	147	Forward	9	ATA/TAG	85.0
*ATP6*	3990–4661	672	Forward	−4	ATA/TAA	74.3
*COX3*	4661–5447	787	Forward	−1	ATG/T	69.5
*trnGly (G)*	5448–5510	63	Forward	0	TCC	84.1
*ND3*	5511–5862	352	Forward	0	ATT/T	76.1
*trnAla (A)*	5863–5925	63	Forward	0	TGC	77.8
*trnArg (R)*	5925–5989	65	Forward	−1	TCG	73.8
*trnAsn (N)*	5989–6053	65	Forward	−1	GTT	73.8
*trnSer1 (S1)*	6054–6120	67	Forward	0	GCT	74.6
*trnGlu (E)*	6121–6186	66	Forward	0	TTC	86.4
*trnPhe (F)*	6190–6256	67	Reverse	3	GAA	79.1
*ND5*	6257–7973	1717	Reverse	0	ATT/T	77.4
*trnHis (H)*	7974–8037	64	Reverse	0	GTG	84.4
*ND4*	8037–9368	1332	Reverse	−1	ATA/TAA	76.4
*ND4L*	9365–9643	279	Reverse	−4	ATG/TAA	79.9
*trnThr (T)*	9646–9709	64	Forward	2	TGT	84.4
*trnPro (P)*	9709–9774	66	Reverse	−1	TGG	75.8
*ND6*	9776–10,273	498	Forward	1	ATA/TAA	81.7
*CYTB*	10,273–11,409	1137	Forward	−1	ATG/TAA	68.1
*trnSer2 (S2)*	11,411–11,479	69	Forward	1	TGA	78.3
*ND1*	11,497–12,447	951	Reverse	17	TTG/TAG	75.8
*trnLeu1 (L1)*	12,449–12,512	64	Reverse	1	TAG	75.0
*rrnL*	12,513–13,778	1266	Reverse	0		78.8
*trnVal (V)*	13,779–13,846	68	Reverse	0	TAC	77.9
*rrnS*	13,847–14,620	774	Reverse	0		76.5
Control Region	14,621–15,527	907	Forward	0		82.1

## Data Availability

The data presented in this study are available in NCBI GenBank (Accession numbers: OP096420 and OP096419).

## Supplementary Materials

The following supporting information can be downloaded at: https://www.mdpi.com/article/10.3390/genes13101881/s1, Figure S1: Predicted stem-loop structures in the control region of *A. glabripennis*; Figure S2: Predicted stem-loop structures in the control region of *D. pseudonotabilis*; Figure S3: Bayesian inference phylogeny of Cerambycidae *s.l.* inferred from mitogenomic data. Numbers at the nodes are posterior probabilities.
