# Supplementary material for: Complete Mitochondrial Genomes and Phylogenetic Positions of Two Longicorn Beetles, Anoplophora glabripennis and Demonax pseudonotabilis (Coleoptera: Cerambycidae)
Source: Genes (Basel). 2022 Oct 17;13(10):1881. doi: 10.3390/genes13101881 (PMC9601424; doi:10.3390/genes13101881)
Supplement: Supplementary file 1 [file genes-13-01881-s001.zip › genes-1926844-supplementary.pdf]

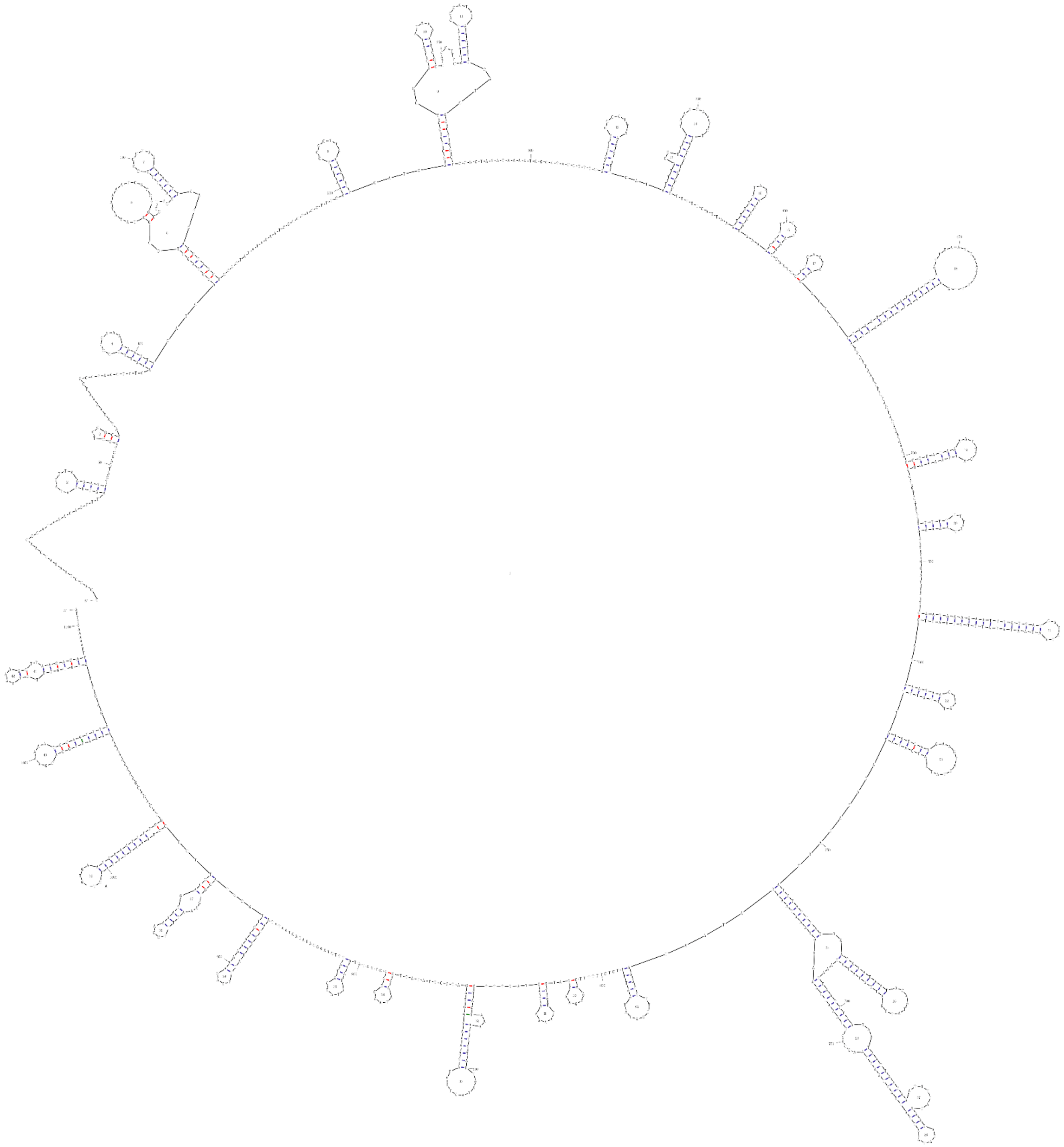

dG = -90.18 13

Figure S1. Predicted stem-loop structures in the control region of *Anoplophora glabripennis*.

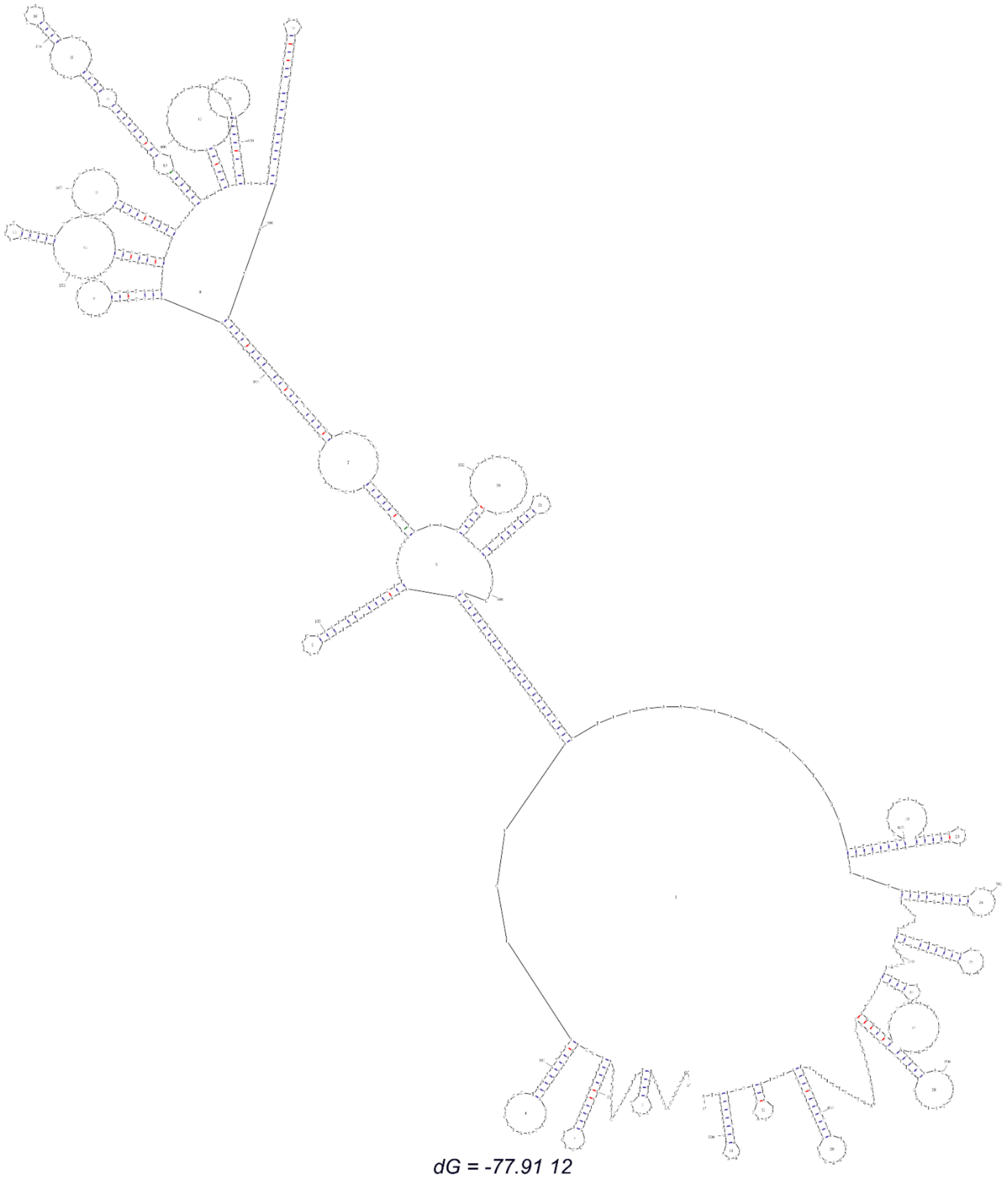

Figure S2. Predicted stem-loop structures in the control region of *Demonax pseudonotabilis*.

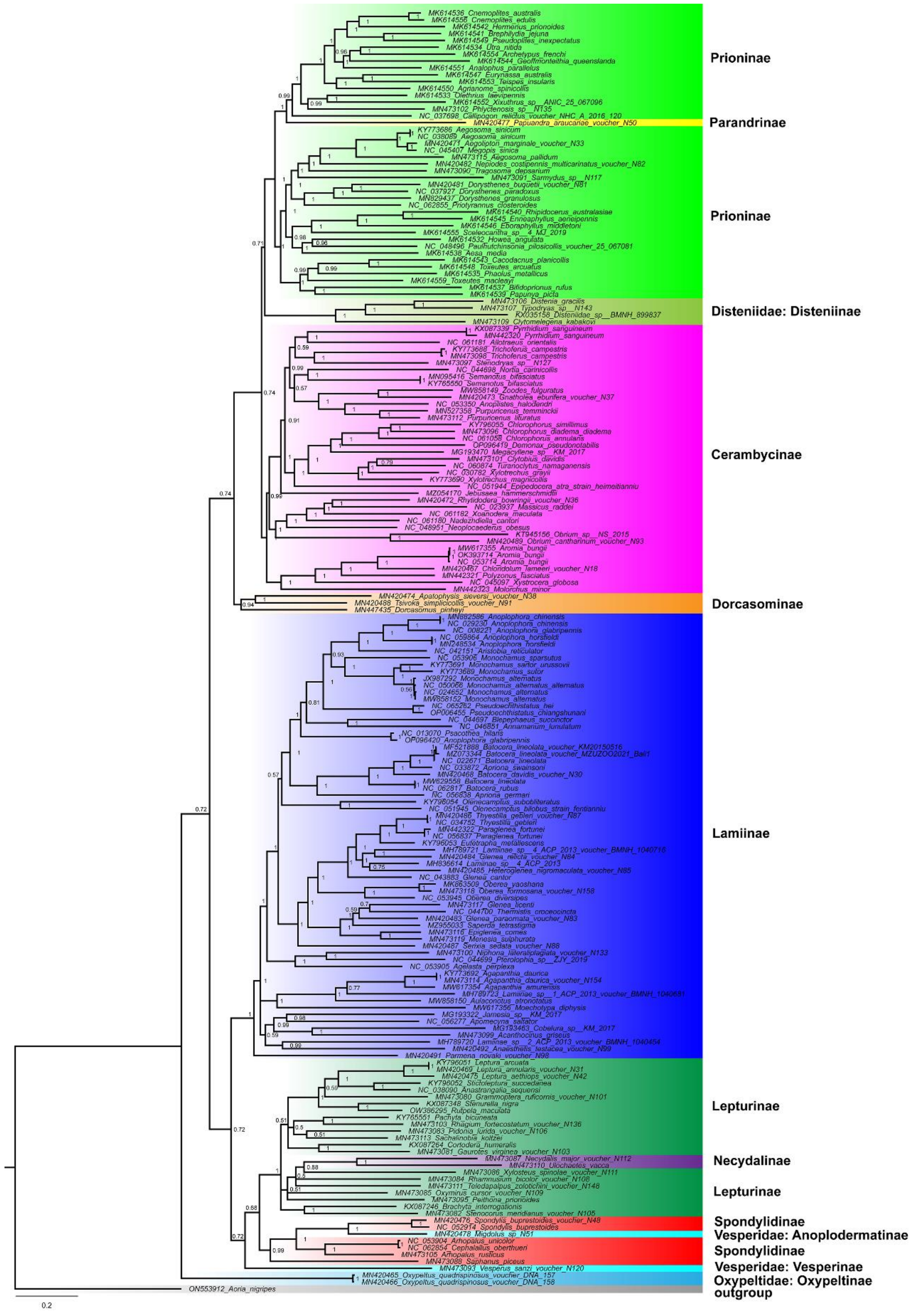

Figure S3. Bayesian inference phylogeny of Cerambycidae *s.l.* inferred from mitogenomic data. Numbers at the nodes are posterior probabilities.
